# Genetic diversity and nutritional analysis of sweet potato [*Ipomoea batatas* (l.) Lam.] genotypes in Abakaliki, Nigeria

**DOI:** 10.1186/s12870-025-06558-y

**Published:** 2025-04-28

**Authors:** Mulugeta Adamu Merga, Issa Zakari Mahaman Mourtala, Wosene Gebreselassie Abtew, Happiness Ogba Oselebe

**Affiliations:** 1 Department of Biotechnology, Mekelle University, Mekelle, Ethiopia; 2 Department of Natural Resources Management, National Institute of Agronomic Research of Niger, Niamey, Niger; 3 Department of Horticulture and Plant Science, Jimma University, Jimma, Ethiopia; 4 Department of Crop Production and Landscape Management, Ebonyi State University, Abakaliki, Nigeria; 5 Department of Plant Science, Bonga University, Bonga, Ethiopia

**Keywords:** Beta-carotene, Flesh color, Genetic diversity, SSR marker, Sweet potato

## Abstract

**Background:**

Sweet potato is an important root crop cultivated in different countries of the world. Its production and productivity are limited by factors such as the use of unimproved local varieties, pests, disease, and drought. To overcome these constraints, diversity in sweet potato genotypes could be a prerequisite for breeding programs. The present study aimed to determine the genetic diversity and nutritional composition of sweet potato accessions. One hundred accessions of sweet potato were collected from Nigeria and Niger for agro-morphological characterization and 50 of them were used for nutritional and molecular analysis. Eleven quantitative traits, six nutritional traits, and ten SSR markers were used for diversity analysis.

**Results:**

Analysis of variance (ANOVA) revealed significant differences (*p* < 0.01) among the sweet potato varieties for all the agro-morphological and nutritional traits studied. Accessions with orange flesh color had higher beta-carotene content compared to those with white, cream, and yellow flesh color. From the molecular diversity analysis, a total of 20 alleles were detected in 50 sweet potato accessions using 10 SSR markers. The average values for Na, Ne, I, Ho, He, and PIC were 2, 1.62, 0.55, 0.40, 0.37, and 0.30 respectively. Cluster analysis based on dissimilarity matrix grouped the accessions into two major clusters. Analysis of molecular variance (AMOVA) revealed 11% variation among the populations and 89% variation within the population, indicating low genetic variation among the populations and high genetic variation within population at *p* < 0.001.

**Conclusion:**

Overall, variability was observed among the studied sweet potato accessions based on agro-morphological, nutritional traits and the SSR markers used. This will help breeding in using these genotypes for further improvement of the studied traits.

**Supplementary Information:**

The online version contains supplementary material available at 10.1186/s12870-025-06558-y.

## Introduction

Sweet potato [*Ipomoea batatas* (L.) Lam.] is an important root crop that belongs to the family of convolvulaceae [1]. It is a perennial plant that grows annually in a temperate climate [1, 2]. It was domesticated in South and Central America, and later introduced to Africa and currently cultivated throughout the humid tropical and subtropical regions of the world [3, 4]. The most frequently observed chromosome number of the sweet potato is 2n = 6 × = 90 and it is an often cross-pollinated plant [5, 6].

According to FAOSTAT, sweet potato cultivation has been reported in more than 110 countries with 93.5 million tons produced in 2023 [7]. China is the leading producer, followed by Malawi, Tanzania and Nigeria. Ethiopia is the tenth largest producer of sweet potato in the world. Its production in China (mainland), Nigeria, Ethiopia, and Niger was 51.4, 4.08, 1.29, and 0.21 million tons, respectively, in 2023 [7].

Sweet potato plays a significant role in the diet of humans and is recognized as the secondary staple food crop in many developing countries [8, 9]. It produces storage roots that are rich in carbohydrates and beta-carotene. They also contain vitamins such as B, C, and E along with minerals like potassium, calcium, magnesium and iron [1, 10]. In addition to its nutritional value, carbohydrates from sweet potato decrease the resistance to insulin by stabilizing the blood sugar levels [11]. Generally, sweet potato is a good candidate for reducing the increasing food insecurity, Vitamin A deficiency, and the under and over-nutrition which mostly occurs in developing countries [12].

Although, sweet potato has high beta-carotene levels [13] and is widely used in developing countries, vitamin A deficiency (VAD) is a public health, causing temporary and permanent eye impairments and increased mortality, especially among children, pregnant, and lactating women. Over 40% of sub-Saharan African children under five years of age have been suffering from vitamin A deficiency [14]. Globally, more than 230 million children have inadequate vitamin A intake, with 13 million of them being affected by night blindness [12].

On the other hand, it has been difficult to reach its maximum yield potential due to several constraints. These main constraints of sweet potato production and productivity include the use of locally unimproved varieties, high incidence rates of pest and disease burden, drought, limited cultivated land and labor shortage [12, 15]. These constraints reduce the vegetative parameter which consequently leads to yield loss. To overcome these constraints, more attention is required in exploiting novel genetic diversity and variability in the local landraces in addition to the different experiments being conducted.

Diversity among germplasm can be assessed using morphological and molecular markers.. Several researchers have reported the phenotypic variability of sweet potato [16–18]. However, these traits are significantly influenced by environmental factors, and many of the traits have quantitative inheritance with partial and dominant expressions [19]. The limitations associated with phenotype-based marker can be addressed through the use of DNA-based molecular markers [20]. Molecular markers like AFLP, RAPD, and SSR have been used for diversity analysis of sweet potatoes [17, 21, 22].

SSR markers are among the good marker for diversity analysis due to their ability to produce informative multiallelic loci and greater genotypic differentiation [23]. They are highly polymorphic markers [24] and abundantly distributed throughout the genome [23]. Though genetic diversity analysis in crops is a prerequisite for breeding programs and preservation of their genetic potential, the genetic diversity analysis of Nigerien sweet potato accessions using SSR markers has not yet been done. Thus, the objective of this study was, to assess the genetic diversity and nutritional composition among sweet potato accessions.

## Materials and methods

### Agro-morphological analysis

#### Description of the study area

The experiment was carried out in the teaching and experimental field of the Department of Crop Production and Landscape Management, Ebony State University (EBSU), Abakaliki, Nigeria. The GPS coordinates of the experimental area are 06^0^ 19′ 47.93^"^N, 08^0^ 07′84.76^"^ E. The annual rainfall ranged from 1700 to 2000 mm. The relative humidity was between 60 and 80%. The minimum, maximum, and mean temperatures were 25^0^C, 38^0^C, and 32^0^C, respectively. The soil was a mixture of 69.4% sand, 16.8% clay, and 13.8% silt soil [25].

#### Plant material

One hundred sweet potato genotypes were used in this experiment. Sixteen improved varieties and 45 advanced lines were obtained from the National Root Crop Research Institute, Umudike, Nigeria, and the other 39 landraces were collected from different agro-ecological regions of Nigeria and Niger.

#### Experimental design and agronomic practice

The experiment was laid out in a 20 × 5 alpha lattice design with three replications. The distance between plots, blocks, and replications is 0.5 m, 1 m and 1.5 m, respectively. Each replication contained 20 blocks and each block had 5 plots. It was planted during the main cropping season of 2021/2022. Ten vines of each sweet potato accession were randomly allocated per plot within each replication. During the growing period management practices such as, fertilizer (NPK 20–10 - 10 application on the second and seventh weeks at 6 g/plot), weeding and pesticide spraying were undertaken as per recommendation.

#### Data collection

The agro-morphological data were recorded according to the descriptor [26]. Data collection for Quantitative (Table S1) and qualitative traits (Table S2) was started aft er 90 days of planting and data on storage root traits were taken after harvesting at 134 days after planting. Data were collected from randomly selected five plants in the middle row of plots.

#### Agro-morphological data analysis

##### Analysis variance

The collected morphological data (quantitative) was subjected to Analysis of Variance (ANOVA) using “Agricolae” package R software [27]. The difference in means between treatments was compared using Duncan’s Multiple Range Test (DMRT) at a 5% probability level. Qualitative data were analyzed using the Shannon diversity index [28].

$$\text{H}^{\prime}= -\sum (\text{piln}(\text{pi})$$

where; pi = the relative abundance of each trait, ln(pi) = the natural logarithm of each abundance, and piln(pi)_=_ the relative abundance of a trait multiplied by its natural logarithm.

The ANOVA model used for analysis was;

$${\varvec{Y}}{\varvec{i}}{\varvec{j}}{\varvec{k}}={\varvec{\mu}}+{\varvec{t}}{\varvec{i}}+\boldsymbol{ }{\varvec{\beta}}{\varvec{i}}+\mathsf{\chi }{\varvec{k}}+{\varvec{y}}{\varvec{l}}+\mathsf{\pi }{\varvec{m}}+{\sum}{\varvec{i}}{\varvec{j}}{\varvec{k}}$$

where

Yijk = response of Y trait from the i^th^ accession, j^th^ replication and k^th^ block, µ = Overall mean effects, ti = Effects of i^th^ level of treatments, β = Effects of the j^th^ level of replication, χk = Effects of k^th^ level of blocks within replications, yl = Effects of l^th^ level of intra block error, πm = Effects of the m^th^ randomized complete block error and $\sum$ijk = is a random error component.

##### Estimations of variance components

To identify genetic variation among the genotypes and to determine environmental as well as genetic effects on the different traits, the following genetic parameters were calculated according to, Singh and Chaudhary [29].

##### Genotypic coefficient of variance

Genotypic coefficient of variation $(\text{GCV}\ \%) =\frac{\sqrt{\sigma {2}\text{g}}}{\text{X}}\text{ X }100$

##### Phenotypic coefficient of variance

Phenotypic coefficient of variation $(\text{PCV}\ \%) =\frac{\sqrt{\sigma {2}\text{p}}}{\text{X}}\text{ X }100$

##### Broad sense heritability

$$H = \sigma 2g / \sigma 2 p$$

##### Genetic advance (GA) and genetic advance as a percentage of the mean (GAM)

Genetic Advance (GA) and Genetic advance as a percentage of the mean (GAM) were calculated according to Johnson et al. [30] at a selection intensity of 5%.

$$\text{GA}=\frac{\text{K}\times \sqrt{{\sigma }^{2}\text{p}}\times {\sigma }^{2}\text{g}}{{\sigma }^{2}\text{p}}$$

where K is constant (2.06 at 5% selection intensity)

$$\text{GAM}(\%)=(\text{GA}/\text{x})\times100$$

where: $$\sigma\text{2g}\;=\;\text{(MSg}\;\text{-}\;\text{MSe)/r,}\;\sigma\text{2e}\;=\;\text{MSe/r}\;\text{and}\;\sigma\text{2p}\;=\;\sigma\text{2g}\;+\;\sigma\text{2e}\;\text.$$

σ^2^_g_ is genotypic variance, σ^2^_p_ is phenotypic variance, σ^2^_e_ environmental variation, MS_g_ is the mean square of accessions, MS_e_ is the mean square of error, r is the number of replications and x is the mean of the traits.

##### Principal component analysis (PCA) and cluster analysis

Principal component analysis (PCA) was used to find out the contributions of the traits to the total variation. Genetic diversity was studied based on various morphological traits using analysis of the cluster among sweet potato accessions. They were analyzed using ‘factoMineR and factoextra” package R. software [31]. The optimal number of the cluster was determined using three statistical tools; gap statistics, average silhouette width, and total within the sum of the square.

### Nutritional analysis

For molecular and nutritional analysis, fifty sweet potato accessions were used (Table S3). The number of accessions was reduced from one hundred to fifty due to the facilities and resource limitations. The fifty accessions were selected based on their status, yield production, and drought tolerance performance from the previous year’s data. Beta-carotene, dry matter, and total sugar (glucose, fructose, and sucrose) contents analysis were carried out at the Department of Biochemistry, National Root Crop Research Institute, Umudike, Nigeria.

#### Determination of beta-carotene content

The beta-carotene content of sweet potato was determined following the methods of Mustapha and Babura. [32]. Ten grams of sample from each macerated sweet potato genotype were measured and mixed with 50 ml of 95% ethanol, then maintained in a70 - 80 °C water bath for 20 min with periodic shaking. After decanting the supernatant and allowing it to cool, the mixture was swirled gently to achieve a homogeneous solution. To obtain a separate layer, 25 ml of petroleum ether was added to the mixture, followed by the addition of cooled ethanol, resulting in two distinct layers.

The absorbance of the final extract (petroleum ether layer) was measured using UV/VIS spectrophotometer at a wavelength of 436 nm. A cuvette containing petroleum ether (blank) was used to calibrate the spectrophotometer to zero point. Reading of samples in cuvette were displayed with figure on the window. The reading was repeated 5–6 times for each sample to record an average reading. The concentration of beta-carotene was calculated using Beer-Lambert’s Law, which states that the absorbance (A) is proportional to the concentration(C) of the pigment, A ∞L (if concentration(C) is constant). A: ECL.

Where: C: concentration of carotene, A: absorbance, E: extinction coefficient, L: thickness of cuvettes (path length) = 1 cm Extinction coefficient of beta-carotene = 1.25 × 104 µg/l.

#### Determination of glucose content

To determine glucose content, 0.5 g of sweet potato was mashed, and 8 ml of HCl (4 N) were added and boiled for 2 h. The extraction was filtered with Whatman No. 1 filter paper and (2 N) NaOH was added to neutralize the filtered solution. Total glucose content was measured by colorimetric method using a commercial enzymatic procedure from spinreacts (Glucose-TR, GOD-PROD, Sant Esteves de Bas, Glrona, Spain). Determination was obtained on a visible spectrophotometer (Genesys Iovis, Thermo coporatiob, Bcilin, Germany) at a wavelength of 505 nm. Data were expressed as grams of glucose per 100 g fresh wt (g/100 g ^−1^ fw) [33]. The percentage of glucose was calculated using the formula

$$\% \text{Glucose }=\text{ Absorbance of standard }-\text{ Absorbance of sample}$$

#### Determination of fructose content

The colorimetric method described by Ojiako and Akubugwo. [34] was used to determine the fructose content as follows; A measured volume of 1 ml of the hydrolysate from the test sweet potato samples was treated with 0.2 ml of dilute indole acetic acid solution in a test tube. Similarly, 1 ml of standard fructose solution (0.05 mg/ml) was treated in the same way in a separated test tube. Then 8 ml of concentrated sulphuric acid (H2SO4) were added to each test tube and warmed in a water bath at 37 ^0^C for 90 min. After cooling to room temperature, their absorbance was read using spectrophotometer at a wavelength of 520 nm with a reagent blank at zero. Then the fructose content was calculated using the following formula:

$$\% \text{Fructose}: 100/\text{w }\times \text{ au}/\text{as }\times \text{ C}/100 \times \text{ vf}/\text{va }\times \text{D}$$

where

w: weight of sample, au: absorbance of sample, as: absorbance of standard, C: concentration of standard (mg/ml), vf: total volume of hydrolysate, va: Volume of hydrolysate analyzed, D: Dilution factor, where applicable.

#### Determination of sucrose content

The sucrose content was determined using the refractometric method as described by Kirk and Sawyer [35]. The temperature of the sample hydrolysate was adjusted to 20 °C, and the refractive index was measured with an Abbe refractometer equipped with a light compensator. To perform the measurement, a smear of the test hydrolysate was placed on the lower prism of the instrument, which was then closed. Light was passed through the instrument, allowing for the formation of a dark background. The telescope was adjusted until a dark image (shadow) appeared at the center of the cross-wire indicator. `

The refractive index was read directly from the screen. This obtained refractive index was used to extrapolate the sucrose content using the international scale table of refractive indices for sucrose solutions at 20 °C. The corresponding sucrose concentration was read directly from this table.

The sucrose content was calculated using the following formula:

$$\% \text{Sucrose }= 100/\text{w }\times \text{ X }\times \text{ D}$$

X: sucrose concentration extrapolated from the standard table.

W: weight of sample hydrolyzed.

D: dilution factor or conversion factor.

#### Nutritional data analysis

The nutritional data were subjected to Analysis of Variance (ANOVA) using “Agricolae” package R software [36]. The difference in means between treatments was compared using Duncan’s Multiple Range Test (DMRT) at 1% probability level. The correlation between agro-morphological and nutritional data was analyzed using “Variability” package R software.

### Molecular diversity analysis

#### Extraction of genomic DNA and SSR primer selection

For the molecular diversity analysis 50 sweet potato accessions were used. The analysis was conducted in Benin Republic, University of Abomey-Calavi, laboratory of Genetics, Biotechnology and Seed Sciences (GBioS). For genomic DNA extraction, young leaves were collected from each genotype and dried using silica gel. The Genomic DNA was extracted from the dried young leaves (0.1 g) using cetyl trimethylammonium bromide (CTAB) method, as described by Doyle and Doyle. [37]. The integrity of genomic DNA was assessed on 1% agarose gel electrophoresis. Ten SSR primers were used for the current sweet potato diversity study (Table 1).

**Table 1:** SSR primers used for the study of genetic diversity in Sweet potato accessions

No	Marker		Forward primers	Reverse primers	Source
1	IBSSR01	GTCACCCATTAACAGCTCTGAGTG		CCATTCGCTGAGCATCTGGAAC	[38]
2	IBS103	CATTATCTCCATCAACTCCTCC		AGCCTGAGAAGAATGGTAATGT	[39]
3	IbU10	GTCTGCGTGTCCGTAGCATA		ATACGCACTTCATATACCGGC	[39]
4	IBR19	GGCTAGTGGAGAAGGTCAA		AGAAGTAGAACTCCGTCACC	[40]
5	IbL16	GTCTTGCTGGATACGTAGAACA		GGGAGAAGTAAGAGAACCGATA	[41]
6	IBS126	TACACATTCGCCTACAGATTTC		GGGAAGACGTAGTAGGTCGTAG	[39]
7	Ib242	GCGGAACGGACGAGAAAA		ATGGCAGAGTGAAAATGGAACA	[42]
8	IBSSR02	GTAACTGAAGTAGTTGGCGAGG		CACTGTGGCTCAATGTAGGG	[38]
9	IbJ263	CTCTGCTTCTCCTGCTGCTT		GTGCGGCACTTGTCTTTGATA	[39]
10	IbJ10a	TCAACCACTTTCATTCACTCC		GTAATTCCACCTTGCGAAGC	[43]

#### PCR amplification and gel electrophoresis

PCR reaction for SSR was carried out in a final volume of 25 μl containing 3 μl genomic DNA, 2.5 μl PCR buffer, 1 μl dNTPs, 2.5 μl of each primer, 0.2 μl Taq polymerase, and 1.25 μl of MgCl2 [17]. The PCR amplifications were carried out in a Gradient MyCycler thermal cycler (BioRad) under the following conditions: 3 min for initial denaturation at 95 °C followed by denaturation for 45 s at 94 °C, 15 s for the annealing temperature at 56–65 °C and 1 min for the extension at 72 °C, with a final 7 min extension at 72 °C. The products were separated by electrophoreses on a 2% agarose gel using a running 1X TBE buffer, at 60 V for 3 h. Finally, the amplified fragments were visualized under a UV transilluminator gel documentation.

#### Analysis of molecular variance (AMOVA) and diversity parameters

Analysis of molecular variance and diversity parameters like major allele frequency (MAF), number of observed alleles (N_a_), Number of effective alleles (N_e_), Shannon’s information index (I), Observed Heterozygosity (H_o_), and expected Heterozygosity (H_e_) were analyzed using GeneAlex version 6.5 software package [44], and polymorphic information content (PIC) of the SSR markers was calculated using power marker version 3.25 software package [45].

$$\text{PIC }= 1 - \sum \text{ pi}2$$

where pi is the frequency of the i^th^ allele.

#### Cluster analysis and principal coordinate analysis (PCoA)

Cluster analysis was carried out using the unweighted pair group method with arithmetic average (UPGMA) and the dendrogram was constructed using DARwin version 6 software [46]. Principal coordinate analysis was also done using GenAlex version 6.5 software package [44].

## Results and discussion

### Agro-morphological analysis

#### Analysis of variance and performance of the genotypes

Analysis of variance showed large variation among the sweet potato varieties for all the agro- morphological traits studied (Table 2). The results revealed highly significant (p < 0.01) differences in all the studied traits, indicating the presence of high variability within the collected sweet potato accessions. This difference may be the result of differences in the collection area (Niger and Nigeria), which leads to differences in genetic makeup. Soil fertility, water and environmental effects could be other factors that increase the degree of variation among the studied sweet potato accessions. This result is in agreement with Alemu and Aragaw. [47], Solankey et al. [48] and Mahaman Mourtala et al. [49] reported significant differences in all the studied traits of sweet potato accessions.

**Table 2:** Analysis of variance (ANOVA) for 11 quantitative traits for 100 sweet potato accessions

**Traits**		**Replication**	**Genotype**	**Error**	**CV%**
	**df**	2	99	141	
**VL**	7292.90		21,463**	2471.20	21.59
**VIL**	0.57		8.51**	1.23	18.05
**VID**	1.62		2.12**	0.73	14.99
**PL**	11.27		91.52**	10.52	16.84
**MLL**	1.95		11.02**	1.85	10.56
**NB**	31.35		3.97**	1.77	26.40
**UBY**	1.93		471.68**	16.35	20.66
**NSR**	0.75		0.92**	0.24	38.74
**SRD**	4.12		1015.92**	132.36	22.33
**SRL**	2.02		37.77**	5.02	17.04
**SRY**	2.21		130.49**	3.81	29.62

*df* Degree of freedom, *CV%* Coefficient of variation in percentage, *VL* Vine Length, *VIL* Vine Internode Length, *VID* Vine Internode diameter, *PL* petiole length, *MLL* mature leaf length, *NB* number of branch, *UBY* upper biomass yield in tone per hectare, *NSR* number of storage root, *SRD* storage root diameter, *SRL* storage root length, *SRY* storage root yield in ton per hectare

^*^: Significant at probability level of 0.05 and **: Significant at probability level of 0.01

Compared to the other traits the upper biomass yield showed the highest range difference (from 71.38 t/ha to 0.83 t/ha), with an average of 17.83 t/ha (Tables 3 and 4). The highest upper biomass yield was recorded for accession Tis- 87/0087 (71.38 t/ha) and the lowest upper biomass yield was recorded for accession EBO/SP4 (0.83 t/ha). The storage root yield ranged widely from 0.11 t/ha to 30.75 t/ha with an average of 6.57 t/ha. The storage root yield observed from this result was greater than that reported by Reddy et al. [50], who reported the highest storage root yield of 9.90 t/ha. Additionally, the highest yield value of the present result was high compared to the yield of the sweet potato in Sub-Saharan Africa (including Ethiopia, Nigeria, and Niger), which ranges from 5 to 25 t/h [14]. Recently, study using Niger and Niegria genotypes reported storage yield from 0.04 to 29.86 t/ha [49]. Thus, in the present study, there were genotypes with high storage root yield characteristics. The highest storage yield was recorded for accession 7 × 12–2 (30.75 t/ha), whereas the lowest yield was recorded for accession Dan Maradi 2 (0.11 t/ha). The high range difference recorded from the upper biomass yield and storage root yield indicates the variation among accessions based on these traits was high compared to the variation due the other traits.

**Table 3:** Mean, maximum, minimum, Estimates of phenotypic (PCV) and genotypic (GCV) coefficients of variations, broad-sense heritability (H), genetic advance (GA) and GA as percent of mean (GAM) for 11 traits of 100 sweet potato varieties

Traits	Mean	Max	Min	σ^2^_p_	σ^2^_g_	σ^2^_e_	GCV (%)	PCV (%)	H (%)	GA	GAM
VL	230.2	791.6	52.3	8802	6331	2471	34.56	40.75	72	139	60.38
VIL	6.02	12.7	1.7	3.66	2.43	1.23	25.89	31.77	66	2.61	43.44
VI D	5.42	9.01	2.19	1.2	0.46	0.73	12.55	20.18	39	0.87	16.08
PL	18.88	37.3	3.5	37.52	27	10.52	27.53	32.45	72	9.08	48.1
MLL	11.56	18.5	5	4.91	3.06	1.85	15.13	19.16	62	2.84	24.61
NB	4.66	11.6	1.3	2.5	0.73	1.77	18.36	33.92	29	0.95	20.47
UBY	0.59	3.34	0.02	0.24	0.13	0.11	61.56	83.06	55	0.55	93.99
NSR	1.18	3.25	0.11	0.46	0.23	0.24	40.37	57.76	49	0.69	58.12
SRD	50.65	100.7	10	426.9	294.5	132.4	33.88	40.79	69	29.37	57.97
SRL	12.08	27	4	15.93	10.92	5.02	27.35	33.05	69	5.63	46.65
SRY	1.27	7.40	0.01	1.56	1.12	0.44	83.33	98.35	72	1.85	145.67

*VL* Vine Length, *VIL* Vine Internode Length, *VID* Vine Internode diameter, *PL* petiole length, *MLL* mature leaf length, *NB* number of branch, *UBY* upper biomass yield in KG per plant, *NSR* number of storage root, *SRD* storage root diameter, *SRL* storage root length, *SRY* storage root yield in KG per plant, *σ*^*2*^_*p*_ phenotypic variance, *σ*^*2*^_*g*_ genotypic variance, *σ*^*2*^_*e*_ environmental variance

**Table 4:** Phenotypic (below diagonal) and genotypic (above diagonal) correlation between 10 quantitative traits of 100 sweet potato accession

Traits	VL	VIL	VID	PL	MLL	NB	UBY	SRD	SRL	SRY
VL		0.77 **	− 0.04	0.12	0.22 *	− 0.34^**^	0.40**	0.23 *	0.42 **	0.21 *
VIL	0.58 **		− 0.24^*^	− 0.14	0.21 *	− 0.39^**^	0.15	0.01	0.19	0.01
VID	− 0.01	− 0.11		0.80 **	0.62 **	0.05	0.37**	0.68 **	0.38**	0.47 **
PL	0.13*	0.01	0.47 **		0.57 **	− 0.21^*^	0.47**	0.37 **	0.27 **	0.27 **
MLL	0.16**	0.24 **	0.34 **	0.48 **		− 0.08	0.37 **	0.47 **	0.31 **	0.38 **
NB	− 0.18^**^	− 0.14^*^	− 0.03	− 0.02	0.03		− 0.3^**^	0.01	0.11	0.01
UBY	0.30**	0.11	0.21 **	0.38 **	0.28 **	− 0.12^*^		0.28 **	0.39 **	0.16
SRD	0.15 **	0.01	0.30 **	0.28 **	0.33 **	0.1	0.23 **		0.53**	0.82**
SRL	0.27 **	0.14*	0.12*	0.18 **	0.21 **	0.06	0.35**	0.46**		0.53**
SRY	0.17 **	0.02	0.30 **	0.22 **	0.31 **	0.02	0.16 **	0.71 **	0.47 **	

*VL* Vine Length, *VIL* Vine Internode Length, *VID* Vine Internode diameter, *PL* petiole length, *MLL* mature leaf length, *NB* number of branch, *UBY* upper biomass yield in tone per hectare, *SRD* storage root diameter, *SRL* storage root length, *SRY* storage root yield

#### Genotypic and phenotypic coefficients of variation

Table 2 shows the genotypic and phenotypic variance as well as the phenotypic coefficient of variation (PCV) and genotypic coefficient of variation (GCV) of all the traits studied among the sweet potato accessions. The phenotypic and genotypic variances for vine length, number of storage root, storage root diameter, and upper biomass yield were high compared to the other traits. The highest and lowest PCV and GCV were 98.35% to 19.16% and 83.33% to 12.55% respectively. According to Sivasubramanian and Madhavamenon [51], the GCV and PCV values greater than 20% are high, those between 10 and 20% are moderate and those < 10% are low. All traits showed high PCV except for mature leaf length (19.16%) which was moderate. The storage root yield (98.35%) had the highest PCV followed by the upper biomass yield (83.06%) and number of storage root (57.76%). Highest PCV of storage root yield following by UBY was reported by recent study on sweet potato [49]. The high PCV value indicates the greater influence of environmental and genetic factors. As, the selection of the desired genotype is impossible depending on only PCV values of the traits, observing GCV values could assist in the identification of the factor.

Higher GCVs were observed for all traits except vine internode diameter (12.55%), mature leaf length (15.13%), and number of branches (18.36%). A greater percentage of GCV was observed for the storage root yield (83.33%) followed by upper biomass yield (61.56%) and number of storage root (40.37%). The higher GCV value of the traits indicates the variation was due to high genetic variation than environmental influences. Generally, from the current results, higher GCV and PCV values were observed for most of the studied traits. This finding highlights the variation in the studied sweet potato accessions because these traits (traits with high GCV and PCV) are more strongly influenced by their genetic make-up than other environmental factors. For all the studied traits, the GCV values were lower than the PCV values which was similar to the findings of Solankey et al. [48] and Mahaman Mourtala et al. [49], indicating the presence of an environmental effect. However, the small difference between PCV and GCV indicates that the influence of the environmental factors was low.

#### Estimation of heritability and genetic advance

The estimates of broad-sense heritability and genetic advance for the traits are presented in Table 3. According to Singh, [52] heritability value greater than 80% is very high, 60–79% is moderately high, 40–59% is medium, and < 40% is low. Higher heritability was noted for storage root yield (72%), vine length (72%) petiole length (72%), storage root diameter (69%), storage root length (69%), vine internode length (66%), and mature leaf length (62%). High heritability in SRY and UBY using Niger and Nigeria accession was also reported recently by Mahaman Mourtala et al. [49]. This Indicates the variation in the accessions due to these traits were from the accessions genetic makeup rather than environmental influence. When variations in specific traits are highly influenced by genetic than environmental factors, selection of the genotype depending on such traits could be better for selection and breeding for the desired character [53]. Moderate heritability was observed for upper biomass yield (55%) and number of storage root (49%), and lower heritability was observed for vine internode diameter (39%) and number of branch (29%). Depending on the values of heritability, the effects of environmental factors on each trait was different. The heritability value for vine length (72%) was high and for the number of branch (29%) was low, which indicates the variation due to number of branchs is highly influenced by environmental factors than the variation from vine length. Similar result was observed by Mahaman Mourtala et al. [49].

The genetic advance as percentage of mean ranged from 16.08% to 145.67% (Table 3). According to Johnson et al. [30], GAM value greater than 20% is high, 10–20% is moderate and 0–10% is low. The majority of the traits studied had a relatively high genetic advance (> 20%) except vine internode diameter (16.08%) which is moderate. Storage root yield (145.67%) had the highest GAM followed by upper biomass yield (93.99%) and vine length (60.38%). Storage root yield and upper biomass yield had higher genetic advance as percentage of means, which were important traits for the selection of the best individuals. Traits with high GAM% value are governed by additive genes and selection would be efficient for the further improvement of such traits [49, 53, 54].

#### Correlation among quantitative traits

Table 7 shows the phenotypic and genotypic correlations among the quantitative traits. Both phenotypically and genetically, storage root yield was positively and significantly correlated with storage root diameter (*r*_p_ = 0.71, *r*_g_ = 0.82) and storage root length (*r*_p_ = 0.47, *r*_g_ = 0.53). In the present study, every contribution to increasing the storage root diameter and storage root length, and selection on the basis of or for these traits led to an increase in storage root yield. The higher genotypic correlation values than phenotypic correlation values indicates the lower influence of environmental factors on their genetic relationships. In other words, the correlation of storage root yield with storage root diameter and storage root length decreased due to environmental influences. The result is in line with Ochieng. [16] and Zakari et al. [54], reported a significant positive correlation between storage root yield with storage root diameter, and storage root length.

Similarly, other traits showed positive and significant phenotypic and genotypic correlation with storage root yield. However, their correlation values were weaker than those of storage root diameter and storage root length. Hence, selection on the basis of storage root diameter and storage root length for increasing storage yield could be better than selection on the basis of other traits. Vine internode length (*r*_p_ = 0.02, *r*_g_ = 0.01) and number of branches (*r*_p_ = 0.02, *r*_g_ = 0.01) had positive but non-significant phenotypic and genotypic correlations. Even though their correlation value was positive, selection on basis of these traits could not improve the storage yield value of the root.

#### Shannon diversity index

In this study, 10 qualitative traits for 100 sweet potato accessions were studied, and their frequency descriptions are presented in Table 5. The similarity and variation of the accessions were observed on the basis of the qualitative traits studied. In terms of plant type, 50% of the accessions were spread, 31% were extremely spread, 17% were semierect, and 2% were erect. Regarding leaf characteristics, like general outline of the leaf, leaf lobe umber, mature leaf color, and mature leaf length, the presence of both variations and similarities were observed.

**Table 5:** Shannon diversity index of qualitative traits

Traits	Category	Freq (%)	H’
	Erect	2	
**Plant Type**	Semi-erect	17	
	Spreading	50	1.09
	Extremely spreading	31	
	Green	22	
	Green with few purple spot	49	
**Predominant vine color**	Green with many purple spot	10	
	Green with many dark purple spots	5	1.49
	Mostly Purple	5	
	Mostly dark purple	6	
	Totally purple	3	
	Green	32	
	Green with purple near leaf	12	
**Petiole pigmentation**	Green with purple at both ends	18	
	Green with purple spots throughout petiole	14	
	Green with purple stripes	11	1.75
	Some petioles purple, others green	2	
	Totally or mostly purple	10	
	Yellow-green	36	
**Mature leaf color**	Green	56	0.89
	Green with purple veins on upper surface	8	
	Absent	67	
**Vine tip pubescent**	Sparse	31	0.71
	Moderate	2	
	None	81	
**Flower Habit**	Sparse	17	0.55
	Moderate	2	
**Traits**	Category	Freq (%)	H’
	Cordate (heart-shaped)	2	
	Triangular	38	
**General outline of the leaf**	Hastate	28	1.35
	Lobed	24	
	Almost divided	8	
	One	37	
	Two	30	
**Leaf lobe number**	Four	1	1.34
	Five	25	
	Seven	6	
	Nine	1	
	White	32	
**Storage root skin color**	Cream	20	
	Pink	20	1.41
	Red	27	
	Purple-red	1	
	1. White	27	
	2. Cream	28	
**Storage root flesh color**	3. Dark cream	11	
	4. Pale yellow	12	1.74
	6. Pale orange	8	
	7. Intermediate orange	12	
	8. Dark orange	2	
**Mean**			**1.23**

H’: Shannon diversity index

High variation was observed in the flesh color and skin color of stored roots. A total of 28% and 27% of the accessions had cream and white storage root flesh color respectively. The accession with orange flesh color (22%) is less compared to those white and cream. Beta-carotene (precursors of Vitamin A) content of sweet potato is highly related to storage root flesh color. The genotypes with orange flesh color have high beta-carotene content than others [55].

The shannon diversity index ranged from 0.55 to 1.75 with an average of 1.23. The highest value was associated with petiole pigmentation (1.75) and the lowest value was associated with flower habit (0.55). The highest Shannon diversity index indicates the even distribution of the characters included under that particular trait, under petiole pigmentation there are characters like, green with purple near the leaf, green with purple at both ends, green with purple spots throughout the petiole, green with purple stripes, some petioles purple, others green and totally or mostly purple.

### Nutritional analysis

#### Analysis of variance for nutritional analysis

The mean squares of the total sugar and beta-carotene contents are presented in Table 6. The results revealed a highly significant difference (*P* < 0.01) among the sweet potato accessions, indicating the presence of variation in their nutritional content. This variation may be attributed to differences in environmental factors and genetic makeup among the accessions. The percentage of dry matter content ranged from 20.38% to 41.29%, with a mean of 31.14%. This result is higher than the findings of Kambale. [56], reported dry matter contents ranging from 16.30% to 30.90%, with a mean of 24.83%. Recent study using Niger and Nigeria genotypes reported DMC from 23.83% to 36.34% [54]. The mean percentages for glucose, fructose, and sucrose were 3.12%, 3.7%, and 9.89%, respectively.

**Table 6:** Mean, maximum, minimum, mean squares and coefficient of variation (CV) for nutritional traits of 50 sweet potato accessions

Traits	Mean	Max	Min	MSg	MSe	CV%
GLU	3.12	4.17	1.19	0.65**	0.01	2.78
FRU	3.7	5.26	2.47	1.28**	0.01	0.72
SUC	9.89	12.7	0.72	5.92**	0.65	8.12
BCA	48.02	87.09	10.3	1340.40**	0	0.05
DM	31.14	41.29	20.38	65.88**	0.01	0.14

*GLU* Glucose, *FRU* Fructose, *BCA* Beta-carotene content, and *DM* Dry matter content

^*^: Significant at probability level of 0.05 and **: Significant at probability level of 0.01

#### Beta-carotene content

In this study, the recorded beta-carotene content ranged from 10.3 to 87.09 µg/g with a mean of 48.02 µg/g, which was higher than the one reported by Mahaman Mourtala et al. [25] (5.67 to 71.38 ug/g) but lower than the maximum value reported in the finding of Kambale. [56] who recorded a range of 0 to 120.57 µg/g with a mean of 54.76 µg/g beta-carotene content for the studied sweet potato genotypes. Additionally, the range of this result (10.3 to 87.09 µg/g) was relatively small compared with the reported ranges for orange fleshed sweet potato, which were 132 to 194 µg/g [57] and 44 to 226 µg/g [58]. These finding indicates the orange flashed sweet potatoes typically have high beta-carotene contents [55, 59, 60]. The lower mean beta-carotene content in the present study can be attributed to the limited number of orange fleshed sweet potato accessions used (only 28%) and other environmental factors that may influence the expression of the responsible gene [55, 60, 61].

### Correlations between agro-morphological and nutritional traits

Table 7 shows the phenotypic and genotypic correlations of 10 agro morphological and 5 nutritional traits studied for 50 sweet potato accessions. The storage root yield was not significantly correlated with any of the determined nutritional traits. It had a negative correlation with the beta-carotene content (*r*_p_ = − 0.17, *r*_g_ = − 0.17) and a positive correlation with the dry matter content (*r*_p_ = 0.08, *r*_g_ = 0.08). This non-significant correlation indicates that the improvement in these traits may not be related to the improvement in the yield performance of the crop. The negative correlation between storage root yield and beta-carotene content was similar to that report in Kambale. [56] and Yada et al. [62].

**Table 7:** Phenotypic (below diagonal) and genotypic (above diagonal) correlation coefficient between agro-morphological and nutritional traits

Traits	VL	VIL	VID	PL	MLL	NB	UBY	SRD	SRL	SRY	GLU	FRU	BCA	DM
VL		0.77 **	− 0.04	0.12	0.22 *	− 0.34**	0.40**	0.23 *	0.42 **	0.21 *	− 0.11	− 0.04	− 0.10	0.10
VIL	0.58 **		− 0.24*	− 0.14	0.21 *	− 0.39**	0.15	0.01	0.19	0.01	− 0.11	− 0.07	− 0.06	0.03
VID	− 0.01	− 0.11		0.80 **	0.62 **	0.05	0.37**	0.68 **	0.38**	0.47 **	− 0.02	0.06	− 0.07	0.09
PL	0.13*	0.01	0.47 **		0.57 **	− 0.21*	0.47**	0.37 **	0.27 **	0.27 **	0.15	0.30*	0.19	− 0.19
MLL	0.16**	0.24 **	0.34 **	0.48 **		− 0.08	0.37 **	0.47 **	0.31 **	0.38 **	− 0.20	0.03	− 0.15	0.07
NB	− 0.18**	− 0.14*	− 0.03	− 0.02	0.03		− 0.3**	0.01	0.11	0.01	− 0.09	− 0.22	− 0.23	0.04
UBY	0.30**	0.11	0.21 **	0.38 **	0.28 **	− 0.12*		0.28 **	0.39 **	0.16	0.12	0.26	0.15	− 0.10
SRD	0.15 **	0.01	0.30 **	0.28 **	0.33 **	0.1	0.23 **		0.53**	0.82**	− 0.16	0.06	− 0.22	0.17
SRL	0.27 **	0.14*	0.12*	0.18 **	0.21 **	0.06	0.35**	0.46**		0.53**	0.07	0.17	0.04	− 0.01
SRY	0.17 **	0.02	0.30 **	0.22 **	0.31 **	0.02	0.16 **	0.71 **	0.47 **		− 0.18	− 0.02	− 0.17	0.08
GLU	− 0.10	− 0.09	− 0.01	0.11	− 0.14	− 0.07	0.09	− 0.16	0.06	− 0.17		0.81 **	0.74 **	− 0.66
FRU	− 0.03	− 0.06	0.03	0.26 **	0.02	− 0.12	0.23 *	0.06	0.14	− 0.02	0.81 **		0.73 **	− 0.7
BCA	− 0.09	− 0.05	− 0.04	0.17	− 0.11	− 0.13	0.14	0.2*	0.03	− 0.17	0.73**	0.73**		− 0.87^**^
DM	0.09	0.02	0.06	− 0.16	0.05	0.02	− 0.09	0.16	− 0.01	0.08	− 0.65**	0.69**	− 0.87^**^	

*VL* Vine Length, *VIL* Vine Internode Length, *VID* Vine Internode diameter, *PL* petiole length, *MLL* mature leaf length, *NB* number of branch, *UBY* upper biomass yield in tone per hectare, *NSR* number of storage root, *SRD* storage root diameter, *SRL* storage root length, *YLD* storage root yield in ton per hectare, *GLU* Glucose, *FRU* Fructose, *BCA* Beta-carotene content, and *DM* Dry matter content

All nutritional traits were positively and significantly correlated with each other except dry matter content, which was negatively and significantly correlated with the other traits, (glucose (*r*_p_ = − 0.65, *r*_g_ = − 0.66), fructose (*r*_p_ = − 0.69, *r*_g_ = − 0.70), and beta-carotene content (*r*_p_ = − 0.87, *r*_g_ = − 0.87)). The beta-carotene content was significantly and positively correlated with glucose (*r*_p_ = 0.73, *r*_g_ = 0.74), and fructose (*r*_p_ = 0.73, *r*_g_ = 0.73). The results were in accordance with Kambale. [56], who reported that dry matter content and beta-carotene content had a negative and significant phenotypic correlation (− 0.622).

### Principal component analysis

**Fig. 1:**
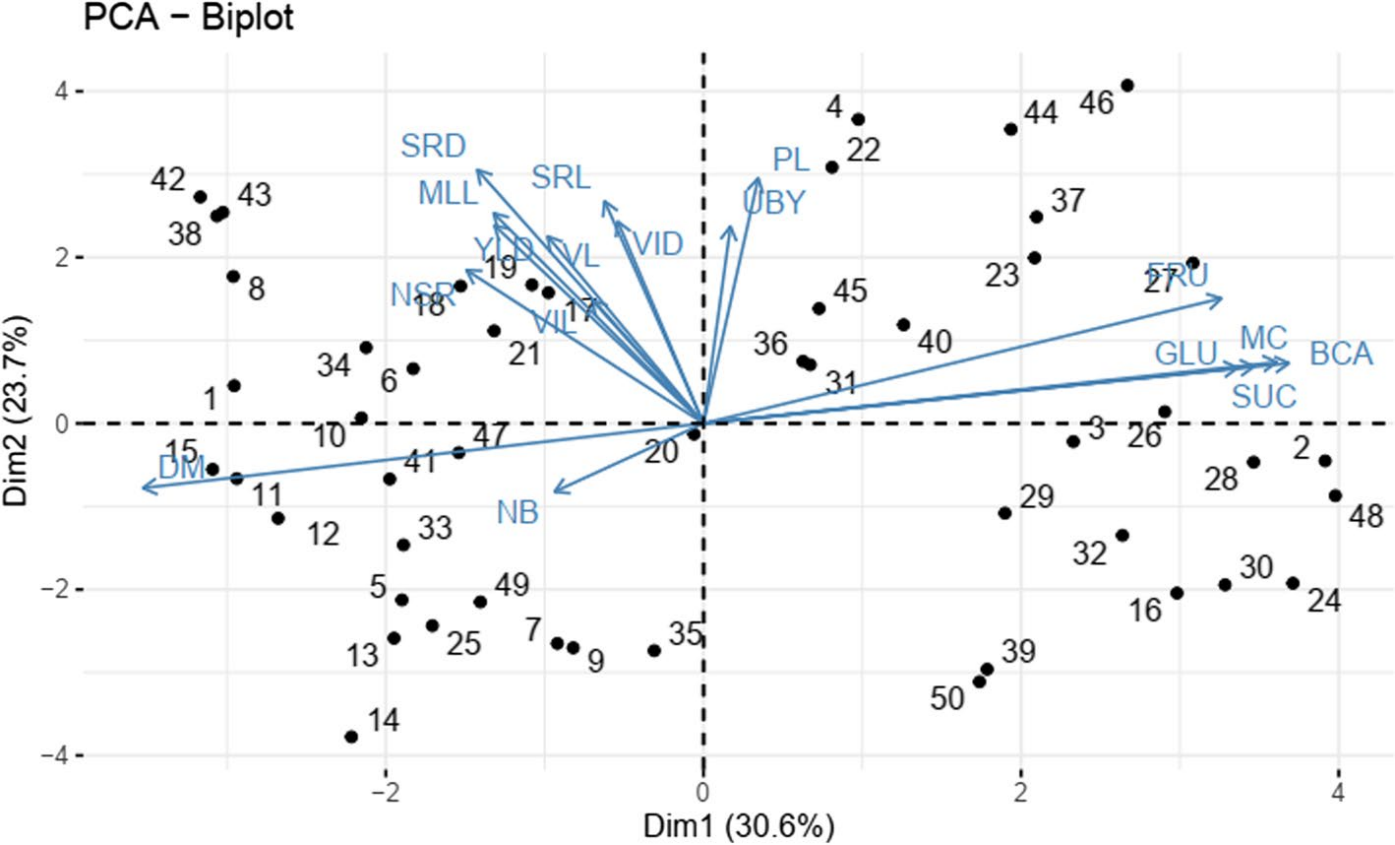
PCA bi-plot showing relationship between accessions and traits (agro-morphological and nutritional). VL: Vine Length, VIL: Vine Internode Length, VID: Vine Internode diameter, PL: petiole length, MLL: mature leaf length, NB: number of branch, UBY: upper biomass yield in tone per hectare, NSR: number of storage root, SRD: storage root diameter, SRL: storage root length, YLD: storage root yield, GLU: Glucose, FRU: Fructose, BCA: Beta-carotene content, and DM: Dry matter content

The Principal Component Analysis (PCA) was performed using agro-morphological and nutritional traits (Table 8). The first two principal components, PC1 and PC2, accounted for 30.60% and 23.70% of the total variation, respectively, with a cumulative variance of 54.30%. The biplot (Fig. 1) illustrates the distribution of genotypes across all quadrants, showing clear separation based on the first two principal components. This dispersion highlights the presence of diversity among the genotypes based on their agro-morphological and nutritional traits.

**Table 8:** Principal component analysis and contribution of the variables

	**Dim. 1**	**Dim. 2**	**Dim. 3**	**Dim. 4**
Eigenvalue	5.195	4.027	1.632	1.516
Variance (%)	30.556	23.687	9.602	8.916
Cumulative (%)	54.243	63.845	63.845	72.761
VL	1.148	7.809	21.897	1.019
VIL	0.583	3.371	33.237	4.367
VID	0.347	9.086	5.590	19.718
PL	0.142	13.407	0.053	17.361
MLL	2.080	9.848	0.972	4.825
NB	1.047	1.042	13.203	0.975
UBY	0.035	8.652	0.213	9.458
NSR	2.653	5.239	13.541	11.895
SRD	2.417	14.338	1.338	6.785
SRL	0.461	11.003	0.886	1.595
YLD	2.063	8.737	8.353	18.185
GLU	13.478	0.688	0.040	0.058
FRU	12.670	3.482	0.100	0.224
SUC	14.324	0.722	0.085	1.255
BCA	16.188	0.807	0.073	0.266
DM	14.839	0.933	0.216	0.900
MC	15.525	0.836	0.202	1.115

### Cluster analysis

**Fig. 2:**
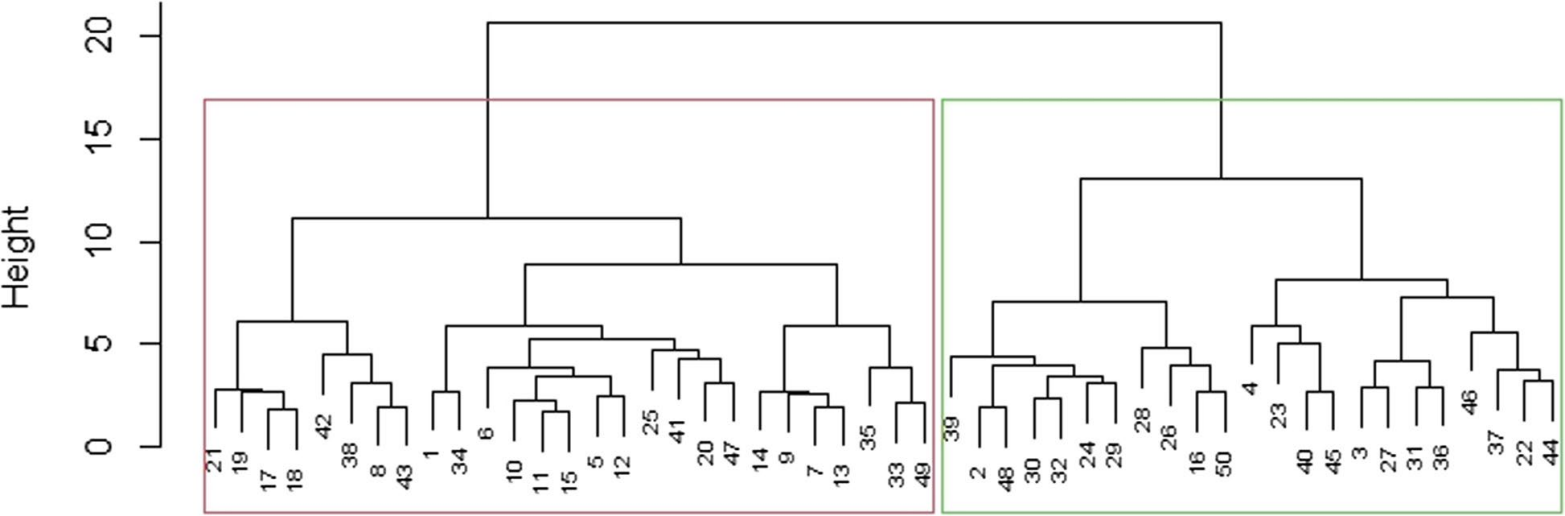
Dendrogram showing the sub grouping of 50 sweet potato accessions based on agro-morphological and nutritional data

The cluster analysis divides 50 sweet potato accessions into two clusters according to their agro-morphological and nutritional traits. As shown in Fig. 2, the first group and second group were composed of 26 (52%) and 24 (48%) accessions respectively. The accessions grouped in cluster one were high in mean storage root yield (7.25 t/ha) and mean dry matter content (35.57%) compared to the accessions in the second cluster which had 4.64 t/ha mean storage root yield and 25.95% dry matter content. On the other hand, accessions in the second cluster were high in mean beta-carotene content (75.18 µg/g) compared to the mean beta-carotene contents (24.89 µg/g) of accessions in the first cluster.

As cluster analysis grouped on the basis of their similarity, the accessions grouped under the same cluster are more similar in their genetic makeup than with the accessions of different cluster. For instance, accessions in cluster one had high mean storage root yield, which means the accessions in the first cluster had almost similar performances in responding to that specific environment compared to the accessions in the second cluster. Generally, the clustering depends on the accession’s genetic makeup which is an input for breeding in identifying their genetic distance.

### Correlation between flesh color, storage root yield and beta-carotene content

**Fig. 3:**
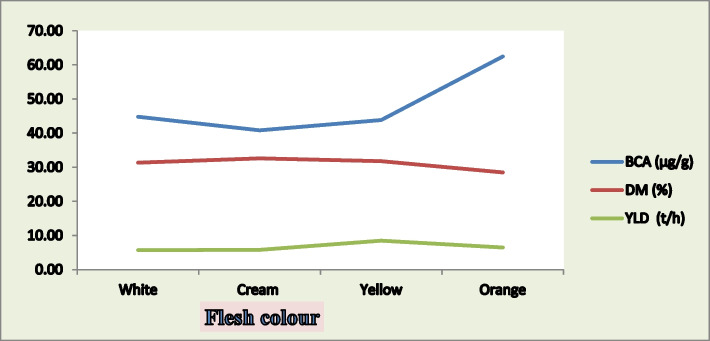
Correlation between sweet potato flesh color with storage root yield (t/h), beta-carotene content (µg/g) and dry matter content (%)

Figure 3 illustrates the relationships between sweet potato flesh color and its storage root yield (t/ha), beta-carotene content (µg/g), and dry matter content (%). The studied sweet potato accessions with white, cream, and yellow flesh colors did differ substantially in terms of storage root yield, dry matter content, and beta-carotene content. However, compared with white, cream, and yellow-fleshed accessions, orange-fleshed accessions presented greater beta-carotene contents and lower dry matter contents. Gurmu and Mekonen. [55] and Mahaman Mourtala et al. [25] reported that sweet potato with an orange flesh color had a lower dry matter content than those with a white flesh color. The observed beta-carotene content was similar to the findings of Kammona et al. [63], who analyzed total carotenoid levels in purple, white, yellow, and orange-fleshed sweet potatoes and reported the highest total carotenoid content in the orange fleshed sweet potatoes. Additionally, Kambale. [56], Gurmu and Mekonen. [55] and Mahaman Mourtala et al. [25] reported that orange-fleshed sweet potatoes contained higher beta-carotene levels than those with other flesh colors.

### Genetic diversity analysis

#### Genetic diversity parameter analysis

For the genetic diversity analysis of 50 sweet potato genotypes, ten SSR markers were used and the genetic diversity parameters are summarized in Table 8. The major allele frequency detected by these markers ranged from 0.54 (IBJ10a) to 0.89 (IBR19) with a mean of 0.73, which reflected allelic variation in the marker loci. Each marker detected two different alleles (Na) per locus, highlighting the genetic diversity present within the sweet potato genotypes studied. The number of effective alleles (Ne) varied between 1.25 and 1.99 with a mean of 1.62. The highest number of effective allele (1.99) was recorded from marker IbJ10a, whereas the lowest number of effective allele (1.25) was observed for IBR19. This finding indicates the existence of allele frequency variation in the marker loci.

Shannon’s information (I) index ranged from 0.35 to 0.69. The highest value of Shannon’s information (I) index was recorded for marker IbJ10a, whereas the lowest value was noted for IBR19 with a mean value of 0.55. The observed heterozygosity (Ho) value varied from 0.10 to 0.92 with a mean of 0.49. The highest observed heterozygosity value was observed for marker IbJ10a (0.92), whereas the lowest value was detected for the marker IbL16 (0.10). The expected heterozygosity (He) value was ranged from 0.20 to 0.50 with a mean of 0.37. Additionally, the highest expected heterozygosity (0.50) was observed for marker IbJ10a whereas, the lowest expected heterozygosity (0.20) was observed for marker IBR19.

##### Polymorphic Information Content (PIC)

As shown in Table 8, the Polymorphic information content (PIC) value ranges between 0.18 and 0.37 with an average of 0.30, which is relatively low compared with the PIC value reported by Nair et al. [42] and Palumbo et al. [64]. Nair et al. [60] used the same SSR primer number (10) on 20 sweet potato genotypes and reported that the PIC value ranged from 0.31 to 0.98 with a mean of 0.76. Similarly, Palumbo et al. [64], reported a PIC value spanning from 0.61 to 0.93 with mean of 0.79 for 11 SSR markers on 22 sweet potato genotypes. However, our results were comparable with the results of Cheng et al. [65], who used 30 SSR primes on 617 sweet potato accessions and reported PIC values ranging from 0.1842 to 0.3593 with an average value of 0.2861. A low PIC value in the present study was observed due to the limited number of alleles (two alleles). According to Botstein et al. [66], a co-dominant locus with a PIC greater than 0.5 is highly informative, a value between 0.5 and 0.25 is reasonably informative, and a PIC value of less than 0.25 implies slightly informative SSR loci for detecting differences among the genotypes. Accordingly markers IbJ10a (0.37), IbL16 (0.37), IBS126 (0.35), Ib242 (0.35), IBSSR02I (0.32) and BSSR01 (0.29) were classified as reasonably informative whereas IbU10 (0.23) and IBR19 (0.18) are slightly informative.

#### Analysis of molecular variance

Analysis of molecular variance (AMOVA) revealed that there was highly significant molecular variance (P < 0.001) within the population (Tables 9 and 10). On the basis of the origin of collection, the accessions were divided into populations (Nigerian and Nigerien populations). The highest percentage (89%) of the variation was attributed to genetic variability within populations, whereas low (11%) variation was observed among the populations, which is similar with the results of Cheng et al. [65], who reported that11% of the total variation was attributed to the variation among groups and whereas 89% was attributed to the variation within groups. Besides, Mahaman Mourtala et al. [67] using SNPs markers reported the highest percentage of the variation within compared to between population based on structure, origin of country, flesh colour, biological status, state of collection and cluster analysis.

**Table 9:** Genetic parameters of the 10 SSR markers used in the study of 50 sweet potato accessions

Markers	MAF	Na	Ne	I	Ho	He	PIC
IBSSR01	0.77	2	1.55	0.54	0.46	0.35	0.29
IBS103	0.83	2	1.40	0.46	0.35	0.29	0.25
IbU10	0.84	2	1.37	0.44	0.32	0.27	0.23
IBR19	0.89	2	1.25	0.35	0.22	0.20	0.18
IbL16	0.57	2	1.96	0.68	0.10	0.49	0.37
IBS126	0.66	2	1.81	0.64	0.67	0.45	0.35
Ib242	0.64	2	1.85	0.65	0.24	0.46	0.35
IBSSR02	0.73	2	1.65	0.58	0.38	0.39	0.32
IbJ263	0.83	2	1.40	0.46	0.35	0.29	0.25
IbJ10a	0.54	2	1.99	0.69	0.92	0.50	0.37
mean	**0.73**	**2**	**1.62**	**0.55**	**0.49**	**0.37**	**0.30**

*MAF* major allele frequency *N*_*a*_ number of observed alleles, *N*_*e*_ Number of effective alleles, *I* Shannon’s information index, *H*_*o*_ Observed Heterozygosity, *H*_*e*_ expected Heterozygosity, *PIC* Polymorphic information content

**Table 10:** Analysis of molecular variance (AMOVA) among and within populations of 50 sweet potato accessions using 16 SSR markers

Source	DF	SS	MS	Est. Var	%	F-statistics	P
Among pops	1	12.01	12.01	0.24	11	Fst = 0.11	0.001
Within pops	98	194.96	1.99	1.99	89		
Total	99	206.97		2.23	100		

The variation within the population can be attributed to the outcrossing nature of sweet potato, despite its predominant propagation via vine cuttings. Although sweet potato is primarily clonally propagated, occasional cross-pollination and seed production contribute to genetic diversity [5, 6, 41]. Even at low frequencies, sexual recombination introduces new genetic combinations, which may become established in populations over time. Additionally, somatic mutations accumulated during clonal growth and the hexaploid nature of sweet potato further enhance within-population variation. Thus, while environmental differences between Nigeria and Niger are minimal due to their shared agro-ecological zone [68], the crop’s reproductive biology and ploidy level play a significant role in maintaining genetic diversity.

The magnitude between and within population differentiation was quantified using F-statistics (Fst), also known as fixation indexes. According to Wright. [69], an Fst value between 0 and 0.05 implies the existence of small genetic differentiation. An Fst value between 0.05 and 0.15 indicates moderate genetic differentiation. Fst values between 0.15 and 0.25 indicate the presence of high genetic differentiation, Fst value > 0.25 indicate the presence of very high genetic differentiation. Thus, the result (Fst = 0.11) revealed moderate genetic differentiation in the studied sweet potato accessions.

#### Clustering analysis and principal coordinate analysis (PCoA)

**Fig. 4:**
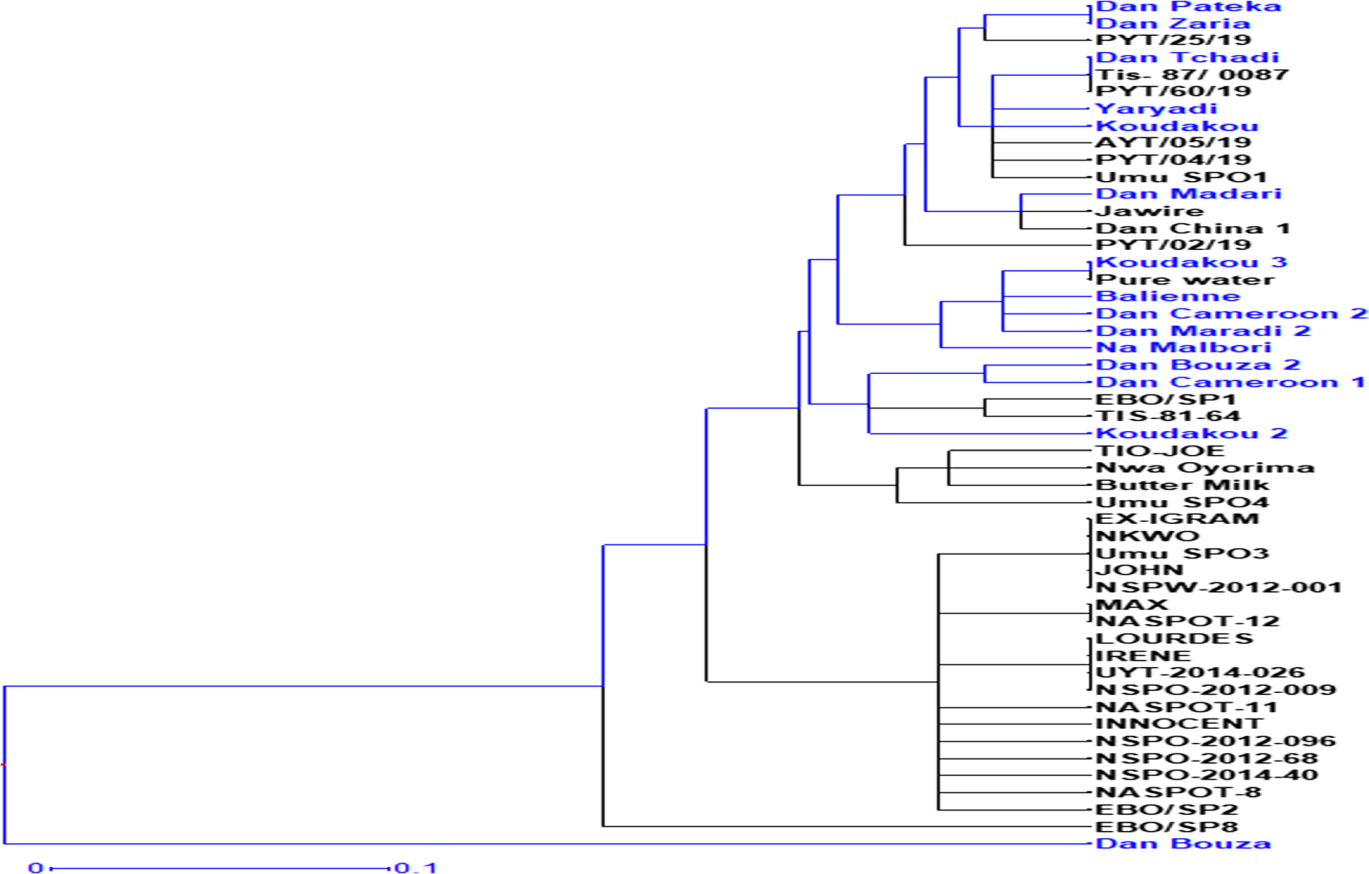
Dendrogram showing genetic relationships of sweet potato accessions based on dissimilarity matrix using SSR markers

Hierarchical clustering grouped the 50 sweet potato genotypes into two major clusters. The first cluster and the second cluster consisted of 98% and 2% of the total population respectively (Fig. 4). The first main cluster contained 49 accessions from both Nigerian and Nigerien populations. The second cluster contains only one genotype (Dan Bouza) from the Nigerien population. The second cluster was divided into different sub-clusters. There were sub-clusters with only the Nigerian population and Nigerien population.

**Fig. 5:**
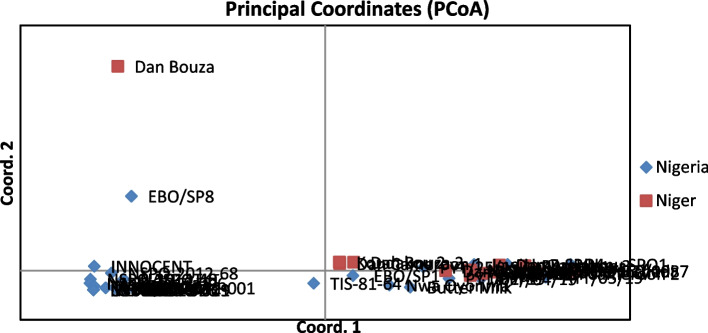
Principal coordinate analysis (PCoA) of sweet potato accessions using SSR markers

Additionally, Principal coordinate analysis (PCoA) revealed that the genotype Dan Bouza was separated from the other accessions (Fig. 5), which supports the result of the cluster analysis. Generally, genetically the Nigerian and Nigerien populations did not clearly separate, indicating the presence of genetic similarity between them. This may be because of the similarities in food, culture, and climate conditions between Nigeria and Niger. Both countries are grouped under the same agro-ecological zone (Sahelian zone) [68]. Thus, the genetic difference between the two populations due to environmental factors was likely minimal.

## Conclusion

This study revealed variability among sweet potato accessions based on agro-morphological traits, nutritional traits, and SSR marker analysis. Storage root yield was positively and significantly correlated with vine length, storage root diameter and storage root length. However, no significant correlation between storage root yield and nutritional traits was detected. Accessions with orange flesh color had significantly higher beta-carotene content compared with white, cream, and yellow flesh colors. SSR markers such as IbJ10a, IbL16, IBS126, Ib242, IBSSR02I, and BSSR0 showed reasonable informativeness based on their polymorphism information content (PIC) values, making them the best markers for genotype identification.

According to the results of the present study, some genotypes were identified for having high yield and high beta-carotene contents and are recommended for further assessment to support the finding. Overall, this study not only contributes to the understanding of sweet potato diversity, it lays the groundwork for future research aimed at optimizing both yield and nutritional quality. The identified accessions represent promising candidates for breeding initiatives focused on enhancing yield and beta-carotene content.

## Supplementary Information

Supplementary Material 1

## Data Availability

The datasets generated and/or analyzed during the current study are available from the corresponding author.
