# Supplementary material for: Genetic diversity and nutritional analysis of sweet potato [Ipomoea batatas (l.) Lam.] genotypes in Abakaliki, Nigeria
Source: BMC Plant Biol. 2025 Apr 28;25:548. doi: 10.1186/s12870-025-06558-y (PMC12036219; doi:10.1186/s12870-025-06558-y)

**Supplementary Figure S1:** Histogram graphs that show agro-morphological and nutritional data distribution


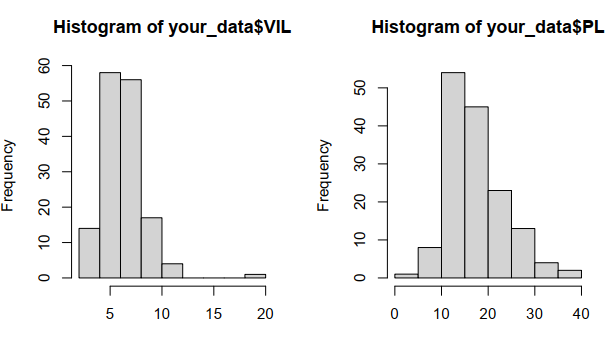


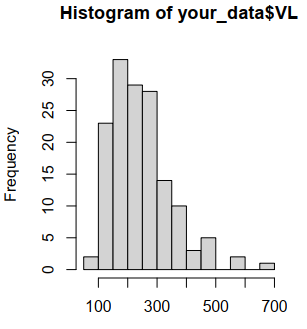

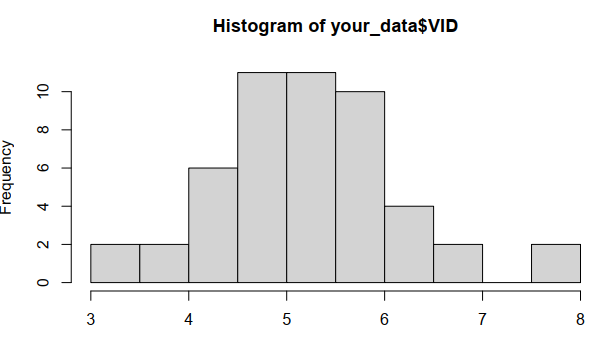


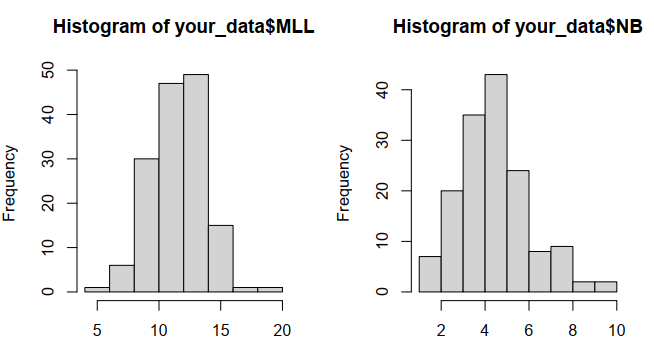


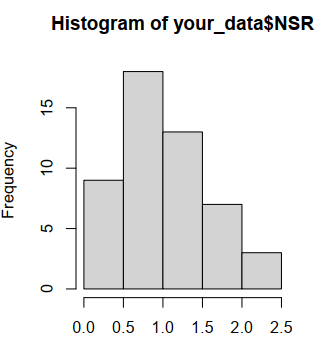

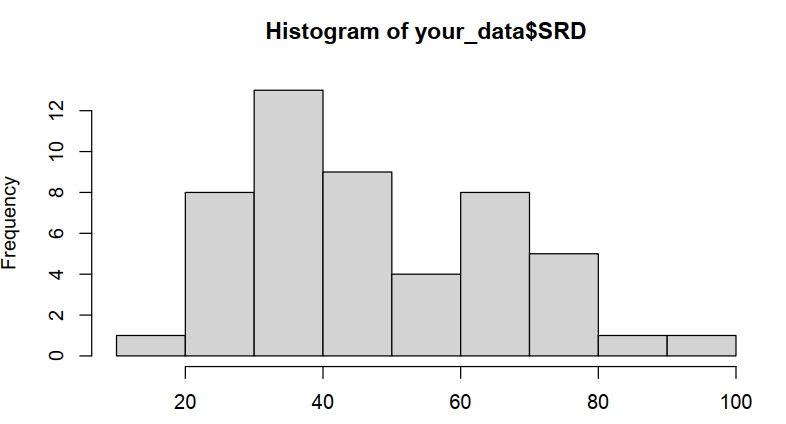


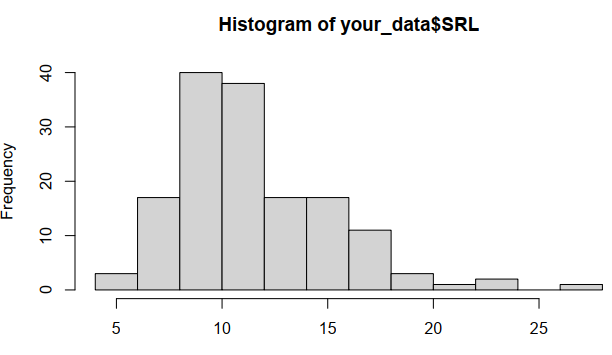

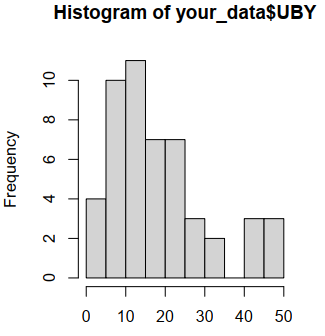


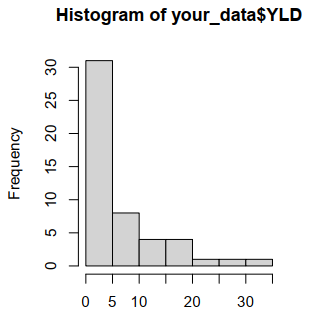


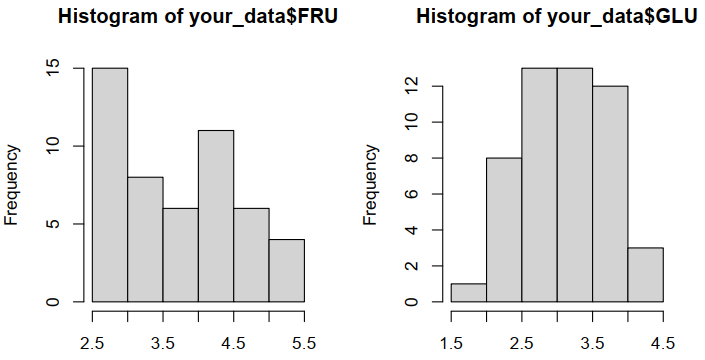


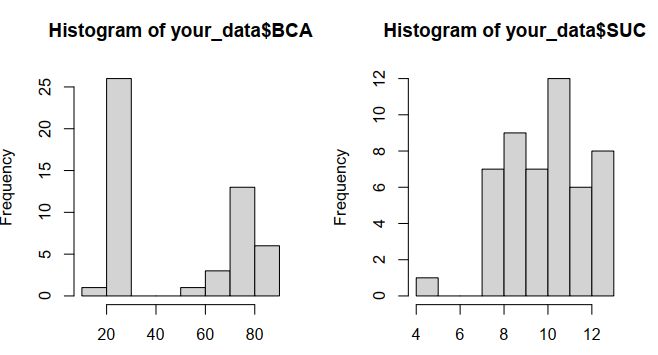


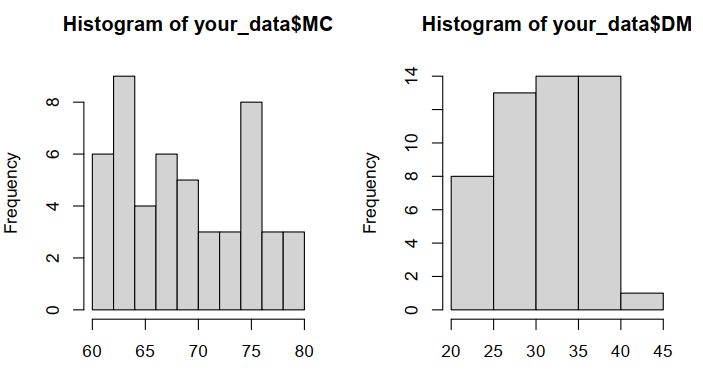

Supplement: Supplementary file 1 — Supplementary Material 1 [file 12870_2025_6558_MOESM1_ESM.zip › Supplementary Figure S1.docx]
